# Structural challenges in the search for mental health services among military police officers: an integrative review 

**DOI:** 10.15649/cuidarte.4131

**Published:** 2025-03-27

**Authors:** Lara Gardênia Bezerra de Melo, Diana Lívia de Sales Lima, Heronildo Almeida Luna Fernandes, Ysabele Yngrydh Valente Silva, Alvaro Micael Duarte Fonseca, Ellany Gurgel Cosme do Nascimento

**Affiliations:** 1 State University of Rio Grande do Norte, Mossoró, Brazil. laragardenia.21@gmail.com State University of Rio Grande do Norte State University of Rio Grande do Norte Mossoró Brazil laragardenia.21@gmail.com; 2 State University of Rio Grande do Norte, Mossoró, Brazil. dianalivia.saleslima@gmail.com State University of Rio Grande do Norte State University of Rio Grande do Norte Mossoró Brazil dianalivia.saleslima@gmail.com; 3 State University of Rio Grande do Norte, Mossoró, Brazil. heronildoalmeida@alu.uern.br State University of Rio Grande do Norte State University of Rio Grande do Norte Mossoró Brazil heronildoalmeida@alu.uern.br; 4 State University of Rio Grande do Norte, Mossoró, Brazil. ysabelevalentin@gmail.com State University of Rio Grande do Norte State University of Rio Grande do Norte Mossoró Brazil ysabelevalentin@gmail.com; 5 State University of Rio Grande do Norte, Mossoró, Brazil. alv.micael@gmail.com State University of Rio Grande do Norte State University of Rio Grande do Norte Mossoró Brazil alv.micael@gmail.com; 6 State University of Rio Grande do Norte, Mossoró, Brazil. ellanygurgel@uern.br State University of Rio Grande do Norte State University of Rio Grande do Norte Mossoró Brazil ellanygurgel@uern.br

**Keywords:** Police, Mental Health, Mental Health Assistance, Stress Psychological, Occupational Health, Policía, Salud Mental, Atención a la Salud Mental, Estrés Psicológico, Salud Ocupacional, Polícia, Saúde Mental, Assistência à Saúde Mental, Estresse Psicológico, Saúde Ocupacional

## Abstract

**Introduction:**

Military police officers daily face stressful and potentially traumatic situations, resulting in a high prevalence of occupational stress and mental comorbidities within this group. Despite this, the pursuit of mental health services by these professionals encounters structural challenges that are still insufficiently discussed.

**Objective:**

To determine the factors that most influence the search for, provision of, and utilization of mental health services by police officers through an integrative literature review.

**Materials and Methods:**

The search for articles was conducted using the following databases: Virtual Health Library (VHL), MEDLINE, Scopus, and Embase. After the selection process, 23 articles were included for analysis in this review.

**Results:**

The main findings indicate that social stigma, lack of knowledge on the subject, and organizational deficiencies are critical aspects that hinder the pursuit of psychological well-being services in the police field.

**Discussion:**

Despite the many barriers to police officers seeking mental health care, it was highlighted that family support and professional development are positively related to help-seeking behavior.

**Conclusion:**

Further research is needed to evaluate mental health programs and better understand the difficulties in implementing these services. Promoting the search for psychological help is essential to improving police officers' health and population's safety.

## Resumen

**Introducción:**

Los policías militares enfrentan diariamente situaciones estresantes y potencialmente traumáticas, resultando en una alta prevalencia de estrés ocupacional y comorbilidades mentales en este grupo. A pesar de esto, la búsqueda de servicios de atención en salud mental por parte de esta categoría de profesionales enfrenta desafíos estructurales que aún no se han discutido lo suficiente.

**Objetivo:**

Determinar los factores que más influyen en la búsqueda, oferta y utilización de los servicios de salud mental por parte de los policías militares mediante una revisión integradora de la literatura.

**Materiales y métodos:**

La búsqueda de artículos se realizó en las bases de datos Biblioteca Virtual en Salud (BVS), MEDLINE, Scopus y Embase. Tras las etapas de selección, se incluyeron 23 artículos para el análisis en esta revisión.

**Resultados:**

Los principales resultados indican que el estigma social, el desconocimiento acerca del tema y las deficiencias organizativas son aspectos cruciales que dificultan la búsqueda de servicios de bienestar psicológico en el ámbito policial.

**Discusión:**

A pesar de los numerosos obstáculos en la búsqueda de atención en salud mental en la policía, se destacó que el apoyo familiar y el desarrollo profesional están relacionados positivamente con la búsqueda de ayuda.

**Conclusiones:**

Es necesaria más investigación para evaluar los programas de salud mental y comprender mejor las dificultades en la implementación de estos servicios. Promover la búsqueda de ayuda psicológica es esencial para mejorar la salud de los policías y la seguridad de la población.

## Resumo

**Introdução:**

Os policiais militares enfrentam diariamente situações estressantes e potencialmente traumáticas, resultando em alta prevalência de estresse ocupacional e comorbidades mentais neste grupo. Apesar disso, a busca por serviços de assistência em saúde mental por esta categoria de profissionais enfrenta desafios estruturais ainda insuficientemente discutidos.

**Objetivo:**

Determinar os fatores que mais influenciam a busca, oferta e utilização de serviços de saúde mental por policiais por meio de uma revisão integrativa da literatura.

**Materiais e métodos:**

A busca pelos artigos foi conduzida nas bases de dados Biblioteca Virtual em Saúde (BVS), MEDLINE, Scopus e Embase. Após as etapas de seleção, 23 artigos foram incluídos para análise nesta revisão.

**Resultados:**

Os principais resultados indicam que o estigma social, o desconhecimento sobre o assunto e as deficiências organizacionais são aspectos cruciais que dificultam a busca por serviços de bem-estar psicológico na área policial.

**Discussão:**

Apesar dos inúmeros obstáculos na procura de cuidados de saúde mental na polícia, foi destacado que o apoio familiar e o desenvolvimento profissional estão positivamente relacionados com a procura de ajuda.

**Conclusões:**

São necessárias mais pesquisas para avaliar os programas de saúde mental e compreender melhor as dificuldades na implementação desses serviços. Promover a busca por ajuda psicológica é essencial para melhorar a saúde dos policiais e a segurança da população.

## Introduction

The daily exposure of police officers at work is strongly associated with physical and mental symptoms, such as depression, anxiety, post-traumatic stress disorder, and suicidal ideation^1^. However, despite the high prevalence of mental comorbidities within this group of professionals, the search for mental health care by police officers is often affected by the prejudice and stigma inherent to institutions and results in significant challenges to the fullness of the emotional well-being of these professionals. In addition to prejudice and stigma, there are other significant barriers to accessing health services, such as lack of knowledge, a distorted view of the effectiveness of treatments, and fear of social and economic repercussions^2^. 

Studies show that psychosocial risk factors in the work environment of military police officers significantly contribute to physical impairment, such as cardiovascular, respiratory, gastrointestinal, dermatological and musculoskeletal manifestations, as well as to the emergence of emotional and cognitive disorders^3^. These factors cover a wide spectrum of elements that affect the health and quality of life of these professionals. At the same time, studies on mental health have consistently shown that the military police duties are perceived as highly stressful. The events these professionals encounter are varied, requiring intervention in situations of intense conflict and tension^4^.

Police officers must discern right from wrong and make decisions during emergency circumstances, often without access to all the information necessary for a sound judgement. Additionally, these professionals face challenges such as rigid hierarchy, excessive bureaucracy, mismatch between available resources and demands, lack of adequate support from the police system, lack of preparation, and hostility on the part of the population in relation to the public image of the police. The constant danger, both during the work day and break, and the threats directed at their families are intrinsic stressors to the profession^5^. 

The combination of these factors results in high stress at work, leading to a significant absence from their functions and high public costs^1^. On the other hand, when satisfied and professionally accomplished, police officers tend to be more supportive of their co-workers and superiors, which can result in even higher levels of well-being and motivation. This feeling of job satisfaction comprises work engagement, a positive affective-cognitive state related to the profession, which involves commitment and alignment of the professional with the environment and work activities^6^. 

This scenario reinforces the urgent need for effective mental health interventions targeted at this population. However, identifying and understanding the elements that hinder police officers' initiative to seek mental health care is a crucial imperative for designing and successfully implementing strategic interventions in mental health for these professionals. In this context, the objective of this study is to identify the structural challenges and facilitators that influence the demand for mental health services by military police officers. 

## Materials and Methods

This study is an integrative literature review with a highly sensitive search strategy. I was conducted using secondary sources, exploratory literature search and a qualitative approach. Initially, the following steps were outlined: identifying the theme and guiding question, establishing criteria for inclusion and exclusion of studies, extracting data from primary studies, evaluating the studies to be included in the review, interpreting the results, and presenting the review/synthesis of knowledge. 

The research question was based on the PICo strategy, where "P" refers to the population/problem of the study (police), "I" refers to the phenomenon of interest (search for services), "Co" refers to the context (mental health). This approach guided the formulation of the question: What are the main challenges that hinder or facilitate the use of mental health services by police officers? 

The survey and analysis of articles were conducted in April 2023. For the selection of studies, databases in health via *Comunidade Acadêmica Federada* (CAFe in Portuguese) access from the Capes Portal Journals: Virtual Health Library (VHL), Medical Literature Analysis and Retrieval System Online Complete (MEDLINE via PubMed), Scopus and Embase were used. The inclusion criteria for selection of articles were based on the language of publication that must include Portuguese, English or Spanish, availability in full text, and approach of the chosen theme (the police officer’s search for mental health services). The exclusion criteria included duplicate articles across databases, review studies, editorials, abstracts, editor’s notes, theses, dissertations, and books. 

The article search was carried out using descriptors contained in MeSH (Medical Subject Heading Terms), DeCS (Health Sciences Descriptors) and Entree to obtain the keywords for the keyword. The following descriptors were chosen: Police, Mental Health and Mental Health Assistance The Boolean operators 'AND' and 'OR' were used to cross-reference the descriptors and their synonyms, resulting in the simplified keyword: police AND mental health AND mental health support. 

**Figure 1:**
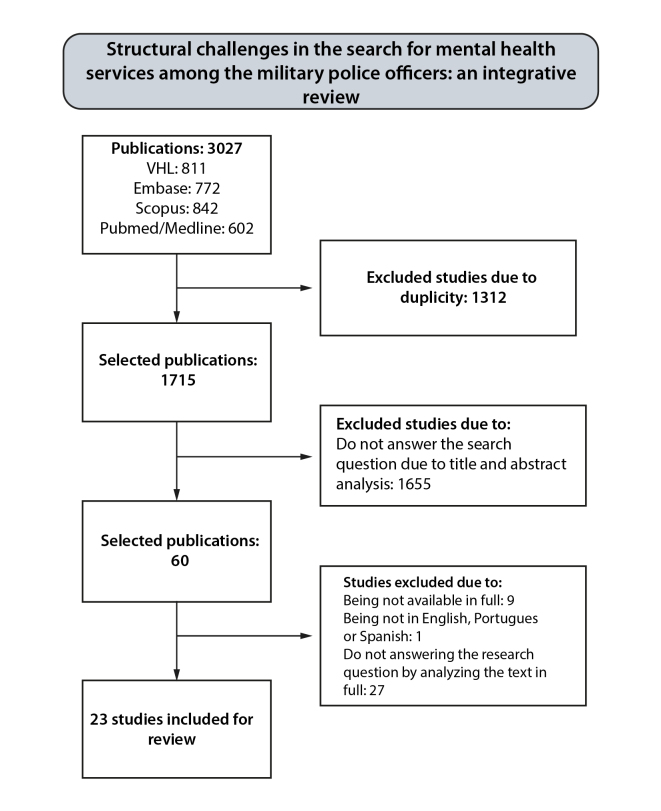
Article selection flowchart

For the selection and analysis of articles, the web application Rayyan (Rayyan Systems Inc.) was used, which allowed the removal of duplicates and selection of articles of interest based on the previously determined inclusion and exclusion criteria. For the primary analysis, the titles, abstracts and keywords of the articles were read blindly by three independent researchers (N=3027). After this initial review, a fourth researcher was brought in to resolve disagreements and reach a consensus on which articles would be selected for full-text reading. After resolving the disagreements, the articles that would be part of the study results were thoroughly read and analyzed (N=60) using a qualitative instrument developed by Ursi (2005)^7^ for data analysis and tabulation (Figure 1). The complete data collected is available for free access and consultation on Mendeley Data^8^. 

Ethical principles in research were fully upheld during the preparation of this article. As it is an integrative literature review, there was no need to obtain approval from the Research Ethics Committee (REC). 

## Results

The study provided a detailed view of the structural challenges that shape the search for mental health services among military police officers. The main relevant findings are presented below in Table 1, highlighting the outcomes of studies that influence the dynamics of this initiative. 

Based on the combination of descriptors and previously established inclusion and exclusion criteria, 23 articles were selected. The publication years varied from 1975 to 2023, with the largest number of publications occurring in 2020 (30.43%), while in 1977, 2012, 2018, and 2023 only one publication was identified for each of the years. The studies included in this review were conducted in five countries: the United States, Canada, the United Kingdom, China, and Australia, with studies in the United States (60.86%) and Canada (26.08%). All studies were of cross-sectional (100%), with the following methodological distribution: 14 studies used a quantitative approach, 5 studies a qualitative approach, and 4 studies a quali-quantitative approach (Table 1). 

**Table 1:** Profile of articles included in the integrative review

N	Country	Sample (N)	Outcomes
Padilla, (2023)^9^	USA	134 participants	Lack of knowledge about mental health services, lack of time, and fear of social repercussions are barriers to using services, however, discussing interventions makes them more comfortable in participating.
Price et al., (2022)^10^	Canada	11 support guide	There is a need to improve understanding of support in pairs available to Public Service Professionals (PSPs) in Canada.
Daniel; Treece, (2022)^11^	USA	86 participants	Secondary traumatic stress is the leading factor associated with seeking mental health services among law enforcement professionals. Social pressure and social engagement are significant predictors of this behavior.
Kyrona et al. (2021)^12^	Australia	14,868 participants	Police and emergency services employees have higher rates of mental health problems compared to the general population.
DePierro et al. (2021)^13^	USA	13,049 participants	Non-traditional life guards have higher rates of stigma around mental health and encounter more barriers to care compared to police officers who responded to the World Trade Center.
Drew et al. (2021)^14^	USA and Australia	7963 participants	It highlights the complexity of the relationships between stigma, help-seeking and mental health, indicating the need for deeper understanding.
Hofer; Savell, (2021)^15^	USA	48 participants	Police officers were willing to engage in mental health services, despite barriers from structural stigma and a lack of trust in the services.
Smith-MacDonald et al. (2021)^16^	Canada	31 participants	Digital health is a viable care model for military personnel, ex-combatants, and public security professionals.
Burns & Buchanan, (2020)^17^	Canada	20 participants	Participants' level of understanding of the potential psychological impacts of police work and the attitudes of those around them influence their search for services.
Carleton et al., (2020)^18^	Canada	4,020 participants	Spousal support, mental health training, and the nature of the profession are all factors that positively influence access to psychological services.
Ricciardelli et al., (2020)^19^	Canada	33 participants	The idea of being rejected by peers was seen as a significant barrier to communicating about mental health and access to treatment.
Jetelina et al. (2020)^20^	USA	434 participants	The lack of awareness about the impact of work on mental health, together with barriers such as confidentiality and psychologists' misunderstanding of the profession, contributes to the lack of treatment by officers.
Krakauer et al. (2020)^21^	Canada	4,108 PSP	Higher levels of mental health knowledge were associated with lower stigma and greater willingness to seek mental health services.
Tatebe et al. (2020)^22^	USA	258 participants	Trauma centers are ideal and safe places to both screen for PTSD and provide mental health care.
Zhu et al. (2020)^23^	China	5467 participants	The mental health of the police was better than that of the public, except for female police officers in guard positions, who had a worse mental state.
Thoen et al. (2019)^24^	USA	55 agencies	Suicide prevention programs are often not formalized and their offer by the agency influences the well-being of police officers.
Ramchand et al. (2019)^25^	USA	110 agencies	Most agencies want to expand mental health services, but face budget constraints and operational challenges. Smaller agencies can benefit from strategic partnerships.
McDevitt, (2018)^26^	USA	990 participants	While treatment strategies are effective, police officers may persist with PTSD symptoms and difficulty concentrating.
Hyland et al. (2015)^27^	United Kingdom	331 participants	Psychological openness and propensity to seek help are positively linked to intentions to seek counseling, while personality differences play a small but significant role in engaging in counseling services.
Karaffa; Ko, (2015)^28^	USA	248 participants	Public stigma and personal stigma are negatively related to seeking professional psychological help.
Fox et al. (2012)^29^	USA	150 participants	Most police officers with mental health disorders do not access health services due to modifiable factors, such as concerns about the confidentiality of services and lack of guidance and training from health professionals.
Brown et al. (1977)^30^	USA	52 participants	Police officers are receptive to various mental health services, although less enthusiastic about the value of the services than professionals and used the services to improve aspects of their personal and professional lives.
Teese; Van Wormer, (1975)^31^	USA	10 departments	Officers became less resistant and more open as they realized that consultants had understood their perspective, even though there was an underlying suspicion of mental health professionals.

In the quantitative studies, forms, questionnaires and scales were used as tools, specific and non-specific for police officers. Among the specific instruments for police officers, the following stand out: Police Stress Survey^32^, Procedural Justice Scale^33^, Inventory of Attitudes Toward Seeking Mental Health Services^34^, The Police Life Events Schedule^35^ and self-authored questionnaires. Among the non-specific instruments, questionnaires and scales were used: Perceived Stress Scale^36^, Trauma Experience Questionnaire^35^ (TEQ) Structured Interview for PTSD^37^ (SI-PTSD) Clinician-Administered PTSD Scale^38^ (CAPS), PTSD Checklist for DSM-5 (PCL-5) and Posttraumatic Growth Inventory^39^. In the qualitative studies, the most used tool was the semi-structured interview and the report (Table 1). 

The samples analyzed in the studies varied in size and included active and administrative police officers, public safety professionals in general (including police, firefighters, paramedics and first responders, rescue personnel and operational intelligence staff), health professionals working with the mental health of police officers, as well as police agencies and departments (Table 1). 

**Figure 2:**
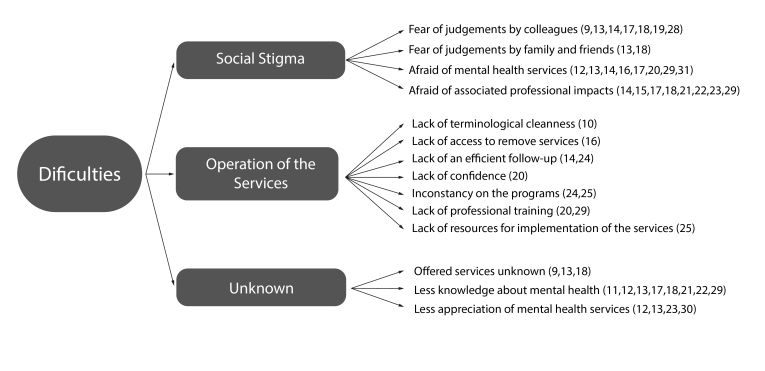
Variables that hinder police officers' access to mental health services

In Figure 2, the variables that most express the structural challenges in searching for mental health services by military police officers are detailed, these being: social stigma (N=17), operation of existing services (N=0) and knowledge of fundamental information to promote adherence to psychological care (N=0). 

**Figure 3:**
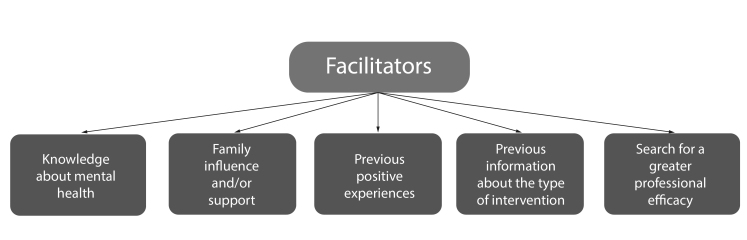
Variables that facilitate the search for mental health services by police officers

The articles also exposed some variables that facilitate the search for mental health services by military police officers, revealing that having knowledge about mental health favors the search (N=1), influence and support of family members (N=2), previous positive experiences (N=1), have prior information about the type of intervention adopted (N=1), and the search for greater professional effectiveness (N=3) (Figure 3). 

These findings provide a solid basis for the formulation of targeted strategies to overcome these structural challenges and promote an environment conducive to the mental health of military police officers. Understanding these elements is essential to guide effective interventions and improve the quality of life of these resolute professionals. 

## Discussion

The analyzed articles showed three main variables directly or indirectly related to the difficulties police officers face to access mental health services, which include social stigma, limited availability of existing services and lack of knowledge, as well as misinformation, often negatively associated with the use of these services. 

The social stigma may stem from structural characteristics inherent to the police institution, based on the stereotypical view of the police officers, historically represented by men, with great physical build and who should never demonstrate emotional fragility. Therefore, for many of the police officers who participated in the studies, using some mental health service for psychosocial support would mean demonstrating mental and emotional weakness^19^, which is different from the image they strive to project, that is, to be completely confident in their own teams^17^. 

In this sense, the reluctance to seek mental health services is partly associated with the fear of demonstrating such vulnerabilities and being seen by their colleagues and superiors as weak or unfit for handling the services involved in police work^14^,^19^. Although in a smaller proportion in relation to the judgment of colleagues, negative perception of family and friends is also of concern. Consequently, these individuals choose to deal with situations of emotional distress on their own, as, even in the family nucleus, they do not feel safe to express psychological and emotional vulnerabilities^13^. 

Another significant barrier, which can also be associated with misinformation, is a lack of confidence in mental health services, both regarding uncertainty about practical benefits and the guarantee of privacy. In this sense, a frequent doubt in police officers’ mindset is whether seeking care will actually bring improvement. Consequently, many of them opt to internalize their feelings and isolate themselves as an alternative, creating a feedback loop of stigma and worsening mental health conditions^17^. 

The distrust in the confidentiality of information shared during consultations is also associated with structural stigma, since the greatest fear among police officers is the potential consequences of information leakage and the impacts on the care^29^,^20^,^13^. In addition, the modality of remote psychological care, a service that has become increasingly common in the face of the needs arising from the COVID-19 pandemic, raises even greater concern about patient safety and privacy, since there is a fear that information shared during the sessions may be saved and/or shared, for instance, in the form of photos or videos^16^. 

Additionally, the concern also extends to negative consequences at work, with possible direct impacts on one's career, such as loss of promotion opportunities, removal from police work, loss of financial stability and even the ability to carry weapons^17^,^13^,^15^. The fear that colleagues in lower, equivalent or higher positions will discover and perceive the individual as fragile or incompetent, along with the fear of possible retaliation from the police agency, are among the main fears hindering the search for mental health services by police officers^9^. 

For female police officers, the stigma inherent in police organization is aggravated by gender issues and the macho man culture. To be seen as equally reliable as their male counterparts, many of them feel that they need to restrain any manifestation of vulnerability, even if this means delaying access to psychological services until they reach a state of significant distress. In general, women are even less likely than men to seek psychological aid^17^. 

Another barrier to accessing mental health services identified in this review was the lack of knowledge about indicators of fragile mental health. In this sense, police officers may confuse the signs and symptoms of occupational stress with normal characteristics of the job, which can contribute to the underestimation or ignorance of a suffering and/or experience of mental health disorder^19^. This lack of early recognition can result in worsening symptoms and reluctance to seek assistance, prolonging psychological distress and negatively impacting the general well-being of this public. 

The lack of information and guidance on the availability of these services also makes it difficult for these supporters to access help, as they often do not know where to go, even when these services are occasionally provided by police departments^9^,^13^,^18^. 

Other factors, more related to organizational issues than to stigma itself, include the lack of formalized mental health services and training offered by police departments^15^,^24^,^25^. Not infrequently, the management of police officers who have dealt with potentially traumatic situations, such as the death of a colleague in the field, does not follow a recognized and proven effective intervention plan, but relies on the personal and subjective decision of a supervisor, so that the director decides whether an officer needs mental health care or not. The lack of a systematic and consistent protocol, with formal guidelines defining the types of incidents that warrant follow-up, makes it difficult to integrate police officers into a mental health support network^15^. 

In addition, while there is a predominant interest by law enforcement agencies and departments to expand mental health support and assistance programs for police, they face persistent budget limitations and ongoing operational challenges, which makes it difficult to continue the treatment of police officers and evaluate the effectiveness of the available services^25^. Without well-established methodological strategies for assessing outcomes and impacts on police officers' mental health, especially in the long term, the body of scientific evidence for many of the approaches used remains scarce. 

Regarding the factors that favor the demand for mental health services among police officers, although most of the studies reviewed did not directly explored these factors, some relevant aspects can be highlighted. Among them, the understanding of fundamental principles related to mental health and the methodologies used in treatment, the ongoing pursuit of improvement to face the challenges of the profession, the accumulation of experience over time, the support received from family and friends, as well as positive previous experiences with the use of such services. 

There is a positive relationship between the mental health knowledge level and the greater propensity to seek professional help, while this relationship is inverse when stigma in involved. Stigma, in turn, decreases as awareness of the benefits of mental health services increases^21^. In this context, prior clarification of the methods and interventions to be used by the services makes police officers more willing to engage in treatment and maintain their participation over time^9^. 

The pursuit of professional advancement was also identified as an important facilitator In the use of mental health services, and once the police officers seek guidance from professionals on how to deal more healthily with the emotional impact of daily professional life, as well as being an empathetic listener able to understand their perspectives^15^,^30^,^31^. This demand becomes even greater the longer the professional’s experience and the positive history with the use of mental health services^31^. 

Another important benefit is the support of family and friends, who are usually the closest group of people able to reorganize the psychological distress of police officers and advise them to seek treatment. In addition, interaction and construction of a support network with loved ones is also important throughout the treatment, because they are often the source of support and engorgement for the professional’s recovery^15^,^23^. 

Despite the relevant information presented in this study, it is important to highlight that the articles included in this review are difficult to compare due the variety of methodologies, scales and variables used in each study. The articles evaluated were from different countries, with unique and distinct realities, requiring the use of diverse scientific study instruments. This review does not analyze the effectiveness of mental health programs offered to police officers due to the comparative difficulties mentioned. Another limitation of this study is the impossibility to follow the daily evolution of the numerous publications on the subject, since it is a current and little-known subject, with new articles being published daily. 

## Conclusions

The results show that social stigma, lack of access to mental health support, and lack of knowledge about this subject are crucial aspects that hinder police officers from seeking psychological well-being services. Conversely, factors such as family support, seeking professional improvement and knowledge of mental health are positively associated with the pursuit of psychological help among police officers. 

However, future research is still needed to evaluate the effectiveness of mental health programs offered, especially those that most encourage police officers to seek help. Additionally, research should consider the difficulties and facilitators presented here when implementing mental health services in the police. It is also worth noting the need for more studies that evaluate the search for mental health services from the perspective of police officers themselves, as they are the main agents in the decision to seek or not seek psychological help and are the most affected by the lack of such support. 

Encouraging the search for mental health services, as well as promoting the development of these services aimed at military police officers are essential steps to improve the health and well-being of the police and, consequently, the safety of the population. In short, the study highlights the need to understand the existing barriers and develop effective strategies for prevention and support of mental health among military police officers, thus enabling the formation of a resilient police force capable of adequately performing its duties, while maintaining quality of life and well-being. 
